# Significance of TNF-*α* and the Adhesion Molecules: L-Selectin and VCAM-1 in Papillary Thyroid Carcinoma

**DOI:** 10.1155/2016/8143695

**Published:** 2016-01-11

**Authors:** Toral P. Kobawala, Trupti I. Trivedi, Kinjal K. Gajjar, Darshita H. Patel, Girish H. Patel, Nandita R. Ghosh

**Affiliations:** Division of Molecular Endocrinology, Cancer Biology Department, The Gujarat Cancer & Research Institute, NCH Compound, Asarwa, Ahmedabad, Gujarat 380016, India

## Abstract

Circulating levels of TNF-*α* and the adhesion molecules L-Selectin and VCAM-1 as well as their expression in the primary tumors of patients with benign thyroid diseases and papillary thyroid carcinoma (PTC) have been determined in this study. The serum levels of TNF-*α*, L-Selectin, and VCAM-1 were significantly higher in patients with both benign thyroid diseases and PTC as compared to the healthy individuals. However, the levels of only TNF-*α* and L-Selectin, and not VCAM-1, were significantly higher in patients with PTC in comparison to those observed in patients with benign thyroid diseases. Further the expression of TNF-*α* and L-Selectin was also significantly higher in the primary tumors of PTC patients, relative to the benign thyroid diseases. The expression of L-Selectin and VCAM-1 significantly correlated with aggressive tumor behavior. In PTC patients, the circulating TNF-*α* levels significantly positively correlated with the levels of L-Selectin, while TNF-*α* immunoreactivity was significantly associated with VCAM-1 expression. Serum TNF-*α* was found to be a significant prognosticator for OS in PTC patients. Overall the results signify that the interaction between TNF-*α* and the adhesion molecules may have a role in thyroid carcinogenesis and understanding this complexity may offer potential therapeutic targets for better management of thyroid cancer.

## 1. Introduction

Thyroid cancer, although being a relatively rare tumour, represents the most frequent endocrine malignancy. According to GLOBOCAN 2012 estimates, it accounts for 2.1% of total new cancer cases and 0.5% of total cancer related death. In India too, there is a significant burden of thyroid diseases with an estimated incidence of thyroid cancer as 1.4% of all new cancer diagnosed with 0.5% mortality rate. It has been estimated that about forty-two million people in India suffer from thyroid diseases [1].

Thyroid cancers can be either follicular cell derived or parafollicular cell derived. The major types of follicular cell derived thyroid cancer include papillary thyroid cancer (PTC), follicular thyroid cancer (FTC), and anaplastic thyroid cancer (ATC), while medullary thyroid cancer (MTC) is the parafollicular cell derived thyroid cancer. Amongst the four histological types of thyroid cancer, PTC and FTC are the differentiated thyroid carcinomas arising from the follicular cells. During the last decades, a rising incidence of thyroid cancer has been noted specifically for PTC, which is the most frequent type, accounting for about 85% of all types of thyroid cancer [2, 3]. The literature repeatedly reports the association between the thyroid cancer and a history of benign diseases. Also accumulating evidences indicate that follicular cell derived thyroid cancer constitutes a biological continuum progressing from the highly curable well-differentiated thyroid cancer to the universally fatal anaplastic thyroid cancer. Although thyroid problems can be readily diagnosed using histologic criteria, very often the pathologist has to face up to thyroid lesions in which the distinction between benign and malignant can be quite subtle. As a result, the decision favouring one or another has clinical consequences and implies different treatment modalities. It implies that, on one hand, there is a need to avoid excessive treatment and psychological discomfort to the patient who has benign thyroid disease or is in the initial stage of differentiated thyroid cancer and, on the other hand, patients with aggressive disease need to be guaranteed effective management right at the initial stage of the disease when it is still curable. Hence, in order to differentiate benign from malignant tumours and in the latter group to distinguish indolent/low risk tumours from aggressive high risk tumours, it is important to decipher the molecular mechanisms underlying thyroid tumourigenesis.

Cytokines are the key mediators of inflammation, which is now being recognized as one of the hallmarks of cancer [4]. Tumour necrosis factor-alpha (TNF-*α*) is a 17 kDa cytokine identified in the late 1970s and is primarily produced by activated macrophages, T lymphocytes, and NK cells [5]. Extensive research so far has revealed various roles of TNF-*α* such as in body development and immunity and in pathological responses such as inflammation, tumour growth, transplant rejection, rheumatoid arthritis, and septic shock [6]. Although TNF-*α* was first identified as a soluble factor capable of inducing tumour necrosis, various mechanisms have been described by which TNF-*α* may promote cancer growth, invasion, and metastasis [7]. Collective evidence has shown that TNF-*α* is a key mediator of inflammation and cancer [8, 9]. Constitutive production of TNF-*α* from the tumour microenvironment is a feature of many malignant tumours and its presence is associated with poor prognosis [10]. At cellular level, TNF-*α* exerts its effects through its receptors to activate distinct signalling pathways that regulate cell survival, proliferation, or death. Consequently, TNF-*α* seems to be having complicated roles in cancer. On one hand, it exerts its anticancer property mainly through inducing cancer cell death, a process that could be used for cancer therapy while, on the other hand, it stimulates proliferation, survival, migration, and angiogenesis in most cancer cells that are resistant to TNF-*α* induced cytotoxicity, resulting in tumour promotion. It also activates vascular endothelial cells and causes endothelial cells to express adhesion molecules for neutrophils, monocytes, and lymphocytes [11].

Cell adhesion molecules mediate homotypic and heterotypic cellular interactions implicated in tumour progression. Makrilia et al. [12] have stated that changes in the expression or function of the cell adhesion molecules have been implicated in all steps of tumour progression, including detachment of tumour cells from primary site, intravasation into the blood stream, extravasation into distant target organs, and formation of secondary lesions. Amongst the five families of adhesion molecules including the cadherins, integrins, selectins, immunoglobulins, and CD44 molecules, leukocyte- (L-) Selectin is a member of the selectin family and vascular cell adhesion molecule-1 (VCAM-1) belongs to the immunoglobulin superfamily.

L-Selectin, also called CD62L, promotes trafficking through binding interactions with carbohydrate ligands on high endothelial venules in lymph nodes or on activated endothelium at sites of inflammation [13]. In a similar way as they facilitate leukocyte arrest in the vasculature and migration to inflamed tissues, the selectins can also mediate tumour cell extravasation and metastasis. In fact, the tumour cells express functional ligands of selectins and interact with selectins expressed on blood vessel walls [14, 15].

VCAM-1, also called CD106, is a 90 kDa glycoprotein, characterized by the presence of seven immunoglobulin domains [16]. The presence of VCAM-1 has been demonstrated on endothelial cells, macrophages, dendritic cells, and surface of cancer cells. The expression of VCAM-1 is known to increase under the influence of proinflammatory cytokines [17]. The cell-cell adhesion leads to clustering of VCAM-1 and the cytoplasmic domain of this clustered VCAM-1 particularly activates Ezrin which in turn activates the PI3K/AKT signaling that suppresses apoptosis and promotes survival signal in the tumour cells [18]. Further, the Oncomine database, consisting of datasets derived from various microarray studies, provides evidence that VCAM-1 is significantly upregulated in various cancer types including brain, breast, ovarian, and esophageal carcinomas [19].

Hence, this study aimed to determine the circulating and tumoural protein expression of TNF-*α* and the adhesion molecules: L-Selectin and VCAM-1 in primary tumours of PTC patients in relation to their expression in patients with benign thyroid diseases. The results were further correlated with the clinicopathological parameters of PTC patients.

## 2. Materials and Methods

### 2.1. Patients

Total 150 untreated patients with pathologically confirmed benign thyroid diseases (*N* = 67) and papillary thyroid cancer (*N* = 83), enrolled between 2008 and 2012 at our institute, were included in this study. None of the patients suffered from any autoimmune disease previously nor were they taking any immunosuppressive or immunomodulant drugs. The patients with benign thyroid diseases comprised those diagnosed with having nodular goitre, multinodular goitre, colloid goitre, adenomatoid goitre, follicular adenoma, Hürthle cell adenoma, and adenohyperplasia. Amongst the 67 benign thyroid disease patients, 45 patients underwent surgery at our institute. The histopathological classification of the tumours was in accordance with the WHO classification. The thyroid cancer patients were staged according to the AJCC/UICC TNM staging system. As in this staging system, patients are staged on the basis of their age (<45/≥45 years); the patients were grouped into younger (<45 years) and elder (≥45 years) age groups. The clinicopathological characteristics of the PTC patients are shown in Table 1. The PTC patients were followed for a period of 4 years or until death within that period. For overall survival (OS) analysis, complete follow-up details were obtained in 92% (76/83) PTC patients. Amongst them, 9% (7/76) patients had persistent disease and hence were not included for the disease-free survival (DFS) analysis. Thus, for DFS analysis, 69/76 PTC patients were included.

### 2.2. Sample Collection

Informed consent was obtained from all patients prior to sample collection and the study was approved by institutional scientific and ethical committees. Pretherapeutic blood samples were collected from all patients as well as from 67 healthy individuals to detect the circulating levels of TNF-*α*, L-Selectin, and VCAM-1. Serum was separated after centrifugation and was preserved at −80°C until analysis. Paraffin embedded tissue blocks of all the patients (who underwent surgery) were retrieved from the histopathology department of our institute. The clinical and histopathological details of the patients were noted from the case files maintained at the medical record department of the institute.

### 2.3. Enzyme Linked Immunosorbent Assay (ELISA) for Determining the Circulating Levels of TNF-*α*, L-Selectin, and VCAM-1

The circulating levels of the TNF-*α*, VCAM-1, and L-Selectin were estimated from the serum samples using specific commercially available ELISA kits (TNF-*α*: Krishgen Biosystems, L-Selectin: Abcam, and VCAM-1: Invitrogen). ELISA assay was performed using the manufacturer's instructions. All standards and samples were run in duplicate and samples with concentration above the highest standard concentration were run after dilution. The mean absorbance of each set of duplicate standards and samples was determined and absorbance of zero standard was subtracted from it. A standard curve was plotted in GraphPad Prism 5 software with concentration of standards on *x*-axis and absorbance on *y*-axis. The unknown concentrations were interpreted by the software from the standard curve generated. The concentrations of the diluted samples were multiplied by the dilution factor to determine the actual concentration.

### 2.4. Immunohistochemistry to Evaluate Tumoral Protein Expression of TNF-*α*, L-Selectin, and VCAM-1

Immunohistochemical staining was performed for detection of tumoural expression TNF-*α*, L-Selectin, and VCAM-1 in primary tumours of PTC patients and in patients with benign thyroid diseases. Briefly, 3–5 *μ*m thick sections were cut from the formalin fixed paraffin embedded tissue blocks using Leica microtome and mounted on APES coated glass slides. The immunohistochemical staining was carried out using MACH4 Universal HRP-Polymer Detection System from Biocare Medicals, USA, as per the manufacturer's protocol recommendations. Rat monoclonal antibody for TNF-*α* (AbD Serotec, MCA1560; 1 : 20), rabbit polyclonal antibody for L-Selectin (Abcam, ab135792; 1 : 50), and mouse monoclonal antibody for VCAM-1 (Santa Cruz Biotechnology, Inc., sc-13160; 1 : 20) were used. Antigenicity was retrieved by heating the sections in 10 mM sodium citrate buffer (pH, 6.0) for 20 mins in a pressure cooker prior to application of the respective primary antibodies.

All the sections were scored independently by two individual observers in a blinded fashion. A semiquantitative Immunoreactive Score (IRS) method of Remmele and Stegner [20] based on staining positivity and staining intensity was implemented. Staining positivity was scored as 0 for no stained cells, 1 for staining in 1% to 10% of cells, 2 for staining in 11% to 50% of cells, 3 for staining in 50% to 80% of cells, and 4 for staining in >80% of cells. The staining intensity was scored as 0 for no staining, 1 for weak/faint staining, 2 for moderate staining, and 3 for intense/dark staining. The IRS score was then obtained by multiplying the staining positivity and the staining intensity and, therefore, theoretically the scores could range from 0 to 12. For statistical evaluation, the median IRS of each molecule in the two subgroups of patients was used as cut-off value to divide the patients into low (≤ median IRS) and high (> median IRS) expression groups, respectively.

### 2.5. Statistical Analysis

The data were analyzed statistically using the Statistical Package for Social Sciences (SPSS) software version 16 (SPSS Inc., USA). Independent samples *t*-test was used to compare the means of circulating levels of analytes between two groups of subjects and also to assess the association of the analytes with the clinicopathological parameters of thyroid cancer patients. Receiver's operating characteristic (ROC) curves were constructed to determine the discriminating efficacy of the circulating markers between healthy individuals and patients. Two-tailed *χ* ^2^ test was used to compare the tumoural protein expressions in benign and carcinoma patients and also to determine the association between protein expression and clinicopathological parameters of carcinoma patients. In case of less than five patients in the cells of 2 × 2 tables, Yate's continuity correction value along with its two-tailed significance was taken into consideration. Correlation between two parameters was calculated using Spearman's correlation coefficient (*r*) method. Univariate survival analysis was evaluated using Kaplan-Meier method and Log rank test was used to analyze difference in survival curves and to assess the prognostic significance of DFS and OS. Multivariate survival analysis was completed using Cox forward stepwise regression model. *P* values ≤ 0.05 were considered to be significant.

## 3. Results

### 3.1. Circulating Levels of TNF-*α*, L-Selectin, and VCAM-1 in Healthy Individuals and Patients with Benign Thyroid Diseases and Thyroid Carcinoma

The circulating levels (mean ± standard error, M ± SE) of TNF-*α*, L-Selectin, and VCAM-1 in healthy individuals and patients with benign thyroid diseases and PTC are depicted in Table 2. It was observed that the circulating levels of TNF-*α*, L-Selectin, and VCAM-1 were significantly higher in patients with benign thyroid diseases, as compared to that in healthy individuals (*P* < 0.001 for each). Further, the levels of TNF-*α* and L-Selectin, but not VCAM-1, were found to be significantly higher in PTC patients as compared to those in patients with benign thyroid diseases (TNF-*α*: *P* = 0.009, L-Selectin: *P* < 0.001, and VCAM-1: *P* = 0.912).

**Figure 1:**
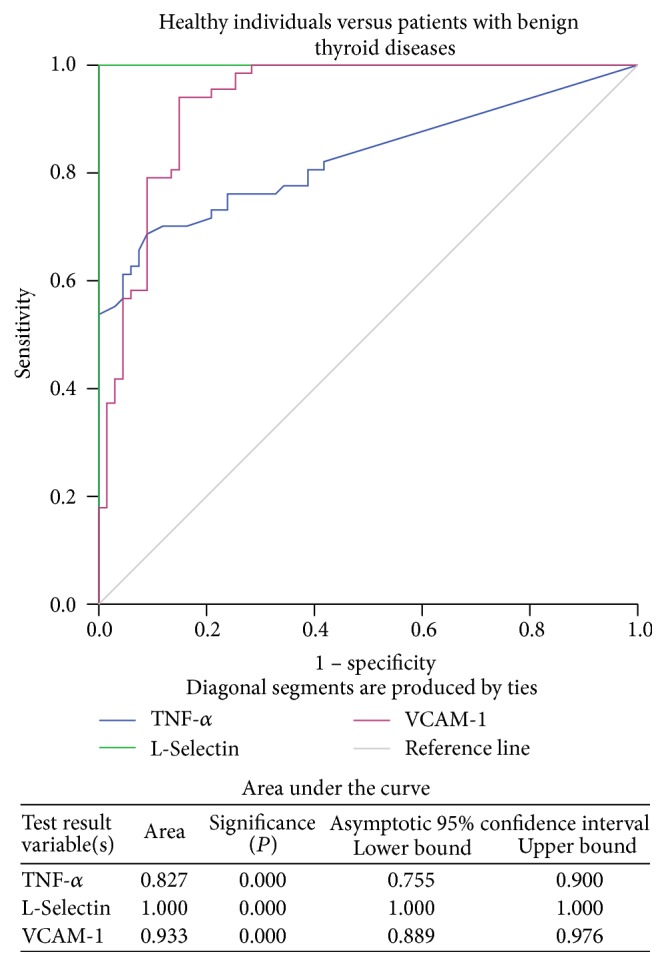
ROC curve for TNF-*α*, L-Selectin, and VCAM-1 in healthy individuals versus patients with benign thyroid diseases.

**Figure 2:**
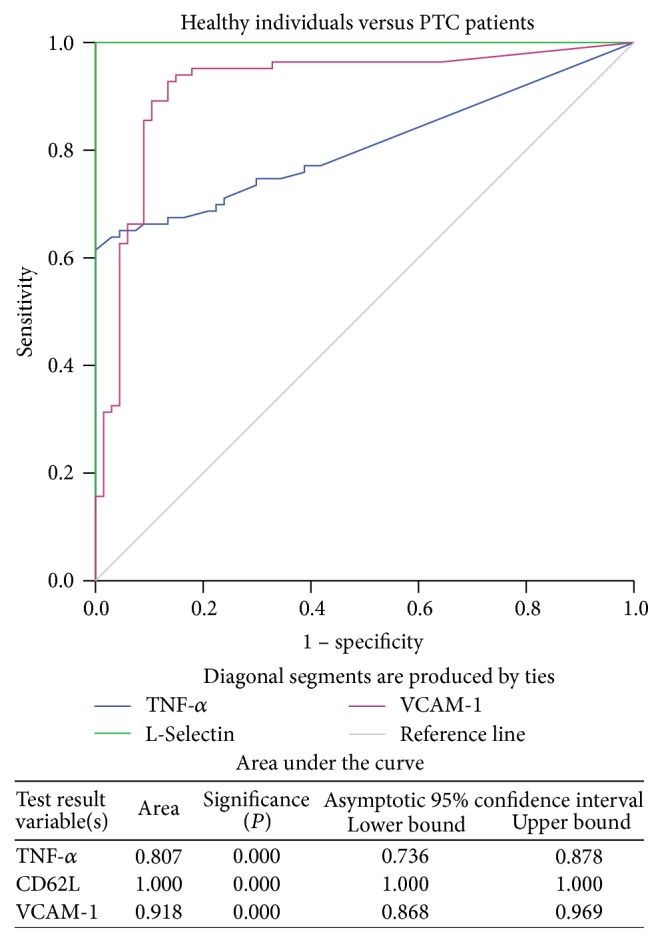
ROC curve for TNF-*α*, L-Selectin, and VCAM-1 in healthy individuals versus PTC patients.

**Figure 3:**
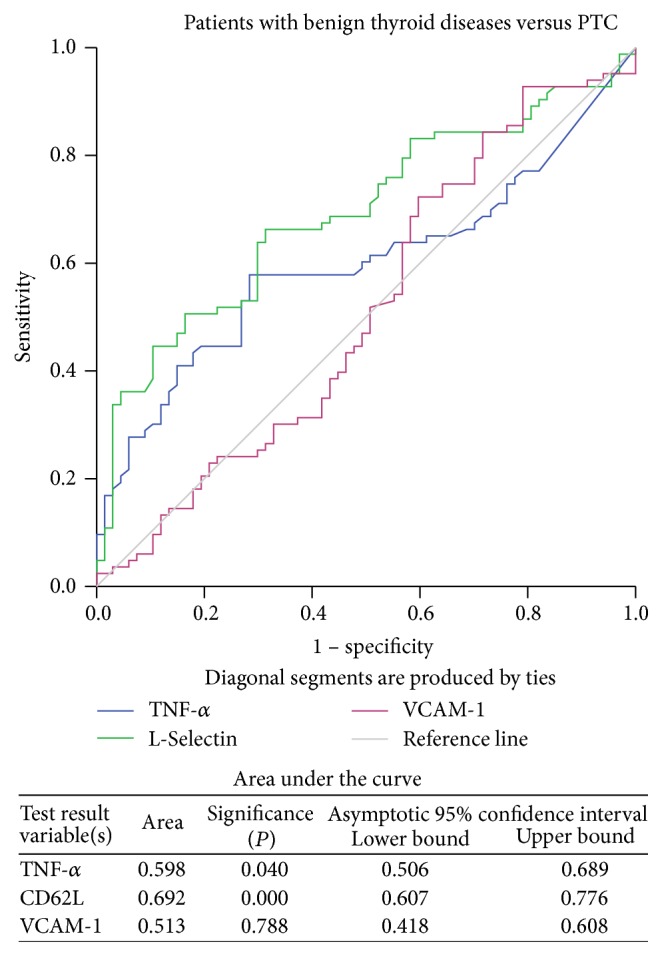
ROC curve for TNF-*α*, L-Selectin, and VCAM-1 in patients with benign thyroid diseases versus PTC.

Further, the ROC curves were generated to reveal the efficacy of these significantly elevated serum cytokine levels in order to differentiate the healthy individuals and patients with different thyroid diseases. The results demonstrated that TNF-*α*, L-Selectin, and VCAM-1 exhibited a good discriminatory efficacy between healthy individuals and patients with benign thyroid diseases (Figure 1) as well as between healthy individuals and PTC patients (Figure 2). Moreover, TNF-*α* (AUC = 0.598, *P* = 0.040) and L-Selectin (AUC = 0.692, *P* < 0.001) but not VCAM-1 (AUC = 0.513, *P* = 0.788) showed good power to discriminate between patients with benign thyroid diseases and PTC (Figure 3).

The correlation of circulating levels of TNF-*α*, L-Selectin, and VCAM-1 with the clinicopathological parameters of PTC patients has been depicted in Table 3. Although the circulating TNF-*α* levels did not show significant correlation with any of the clinicopathological parameters, a trend of correlation was observed with calcification and extrathyroidal extension of tumours. The circulating levels of L-Selectin were significantly higher in PTC patients whose tumours showed haemorrhagic area as compared to those in whom haemorrhagic area was absent (*P* = 0.010) and VCAM-1 levels were significantly higher in patients having bilateral tumours than those with unilateral tumours (*P* = 0.037). Further, no substantial interaction of serum levels of the two adhesion molecules was observed with the remaining clinicopathological parameters (Table 3).

### 3.2. Tumoral Protein Expression of TNF-*α*, L-Selectin, and VCAM-1 in Patients with Benign Thyroid Diseases and PTC

**Figure 4:**
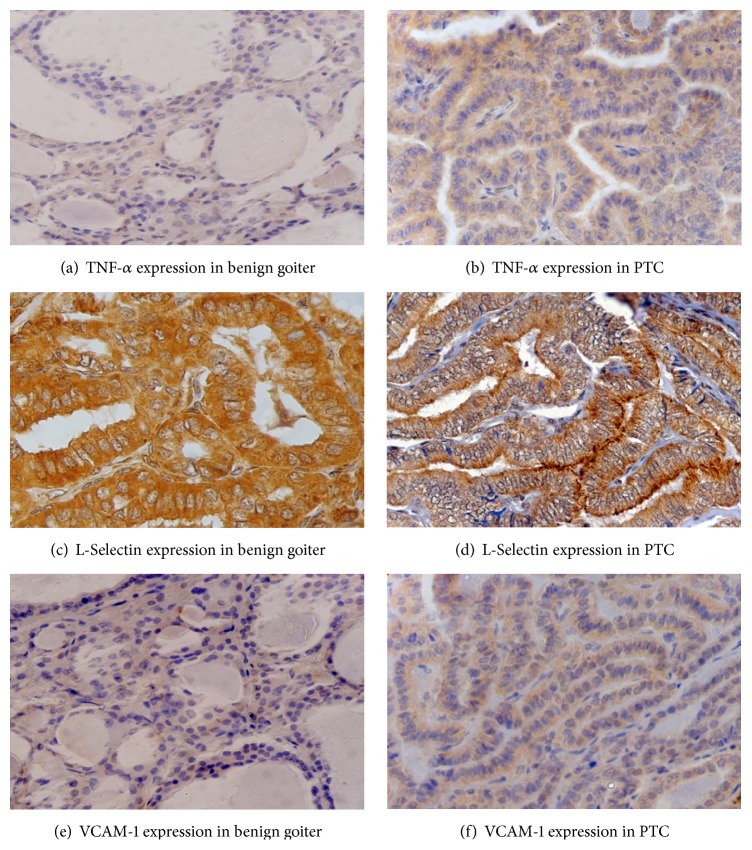
Representative staining patterns of the protein expressions of TNF-*α*, L-Selectin, and VCAM-1 in primary tumours of patients with benign thyroid diseases and PTC.

The expression of TNF-*α* and VCAM-1 was observed in the cytoplasm of the thyroid follicles, while cytoplasmic and membranous staining was observed for L-Selectin. TNF-*α* expression was observed in 67% (30/45) of patients with benign thyroid diseases with median IRS-3 and in 89% (74/83) of PTC patients and IRS-3 emerged as the median score. L-Selectin immunoreactivity was observed in 82% (37/45) of benign thyroid disease patients, and the median score was IRS-6. Further, 51% (23/45) of patients with benign thyroid diseases showed expression for VCAM-1 having IRS-1 as the median score. On the other hand, 99% (82/83) of the PTC patients exhibited L-Selectin expression and the IRS-9 was observed as the median score and VCAM-1 expression was positive in 84% (70/83) of the tumours with median score as IRS-2. The representative staining patterns of the protein expressions are shown in Figure 4.

**Figure 5:**
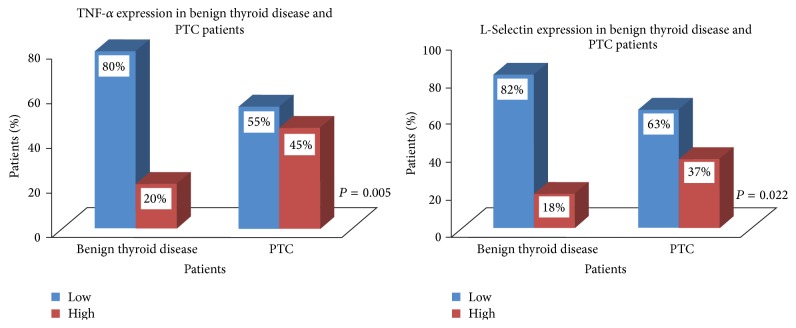
Expression of TNF-*α* and L-Selectin in patients with benign thyroid disease and PTC.

The immunoreactivity of TNF-*α* and L-Selectin was found to be significantly high in PTC patients as compared to the benign thyroid disease patients (TNF-*α*: *χ* ^2^ = 7.657, *r* = +0.245, *P* = 0.005 and L-Selectin: *χ* ^2^ = 5.276, *r* = +0.203, *P* = 0.022) (Table 4, Figure 5). On the other hand, the incidence of expression of VCAM-1 in PTC patients was not significantly different from that in the benign thyroid disease patients (*χ* ^2^ = 1.831, *r* = +0.120, *P* = 0.179) (Table 4).

Moreover, when correlated with the clinicopathological parameters of PTC patients, the TNF-*α* expression was significantly positively correlated only with presence of calcification (*χ* ^2^ = 5.706, *r* = +0.262, *P* = 0.017). Further, significant positive correlation of L-Selectin expression in PTC patients with larger tumour size (T3 + T4) (*χ* ^2^ = 7.955, *r* = +0.310, *P* = 0.004) and presence of extrathyroidal extension of tumours (*χ* ^2^ = 12.120, *r* = +0.382, *P* < 0.001) as compared to the respective counterparts was observed. VCAM-1 expression showed a significantly positive correlation with larger tumour size (T3 + T4) (*χ* ^2^ = 6.219, *r* = +0.274, *P* = 0.012), presence of lymph node metastasis (*χ* ^2^ = 3.971, *r* = +0.219, *P* = 0.047), and extrathyroidal extension of tumours (*χ* ^2^ = 11.350, *r* = +0.370, *P* = 0.001) as compared to their respective counterparts. On the other hand, a significant inverse correlation of VCAM-1 expression was seen with the presence of multifocality of tumours (*χ* ^2^ = 4.040, *r* = −0.221, *P* = 0.045). Apart from these, expression of none of the two adhesion molecules exhibited a significant association with the rest of the clinicopathological parameters (Table 5).

A significant inverse correlation was observed in the circulating TNF-*α* levels and TNF-*α* protein expression in the primary tumour tissues of PTC patients (*r* = −0.254, *P* = 0.021). However, statistical data analysis did not express any significant correlation between the circulating adhesion molecules and their respective protein expressions in PTC patients. Further, the circulating levels of TNF-*α* showed a significant positive correlation with the circulating levels of L-Selectin (*r* = +0.303, *P* = 0.005), while the protein expression of TNF-*α* (*r* = +0.347, *P* = 0.001) showed a significant positive correlation with VCAM-1 expression in the primary tumours of PTC patients (Table 6).

**Figure 6:**
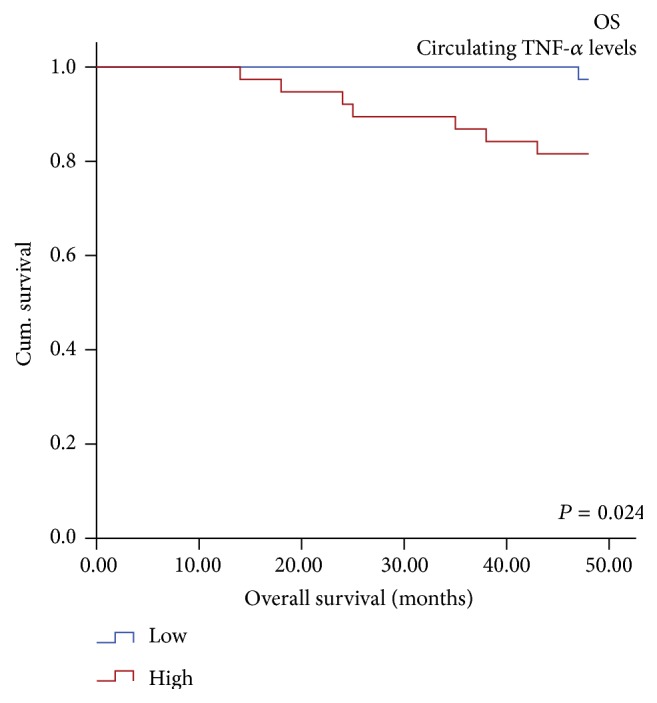
Significantly reduced OS observed in PTC patients with high levels of serum TNF-*α* as compared to its counterpart.

In univariate survival analysis, the circulating levels of none of the studied parameters could predict DFS in PTC patients (Table 7). Further, Log rank test indicated that besides age, gender, metastasis, stage, and multifocality, serum TNF-*α* (Log rank = 5.12, df = 1, *P* = 0.024) was a significant prognosticator for OS in PTC patients (Table 8). The Kaplan-Meier survival curve indicated significantly reduced OS in PTC patients having higher levels of serum TNF-*α* than those having lower circulating TNF-*α* levels. The event of death was significantly higher in PTC patients with high serum TNF-*α* levels (18%, 7/38) than in patients with low serum TNF-*α* levels (3%, 1/38) (Log rank = 5.129, df = 1, *P* = 0.024) (Figure 6). However, in multivariate analysis, serum TNF-*α* lost its significance. Moreover, the circulating levels of L-Selectin and VCAM-1 were not able to predict OS in the PTC patients.

**Figure 7:**
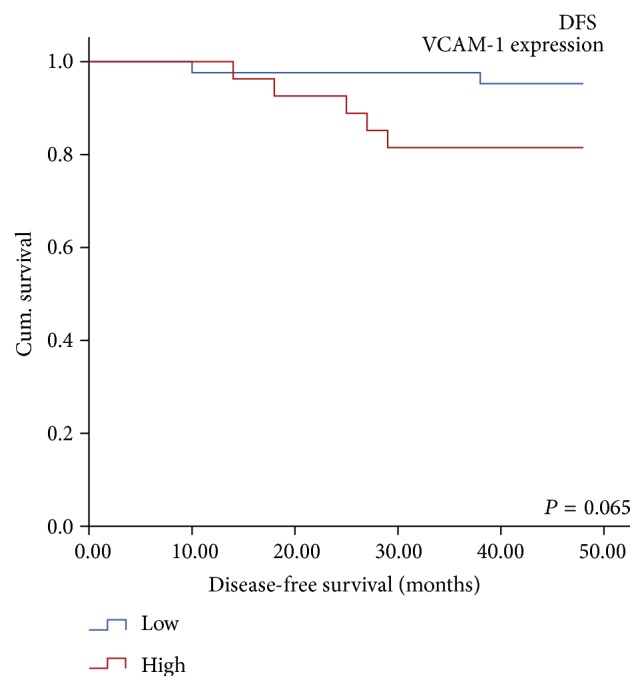
Reduced DFS observed in PTC patients with high VCAM-1 expression as compared to those having low VCAM-1 expression.

The tumoral expression of none of the studied proteins was able to predict DFS or OS, in the PTC patients. The Kaplan-Meier survival analysis demonstrated higher incidence of disease relapse in patients with high VCAM-1 expression (18%, 5/27) as compared to those with low VCAM-1 expression (5%, 2/42). However, this difference was not statistically significant (Log rank = 3.415, df = 1, *P* = 0.065) (Table 7, Figure 7).

**Figure 8:**
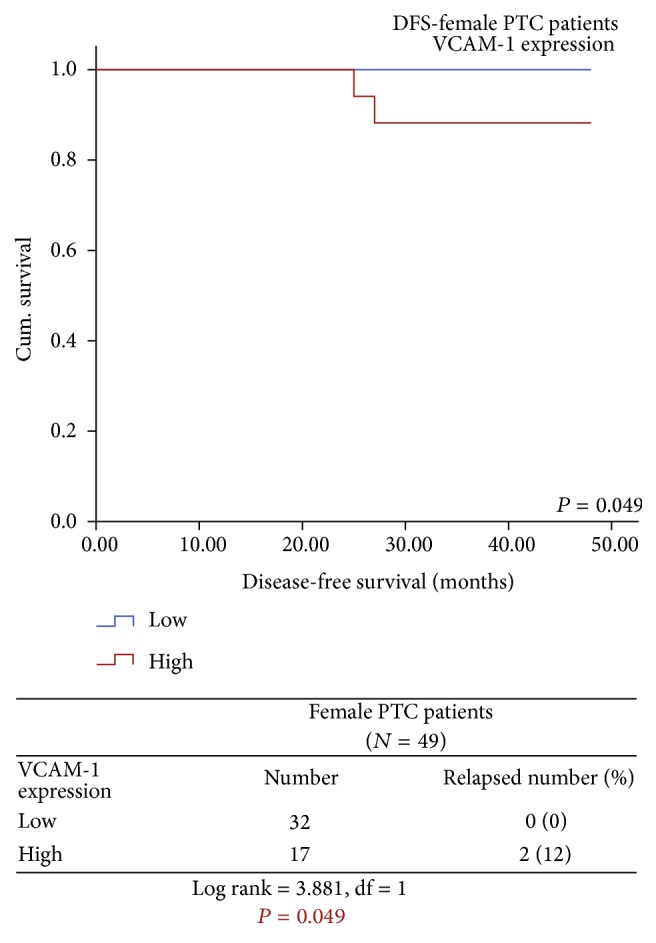
Significantly reduced DFS observed in female PTC patients with high VCAM-1 expression as compared to those with low VCAM-1 expression.

**Figure 9:**
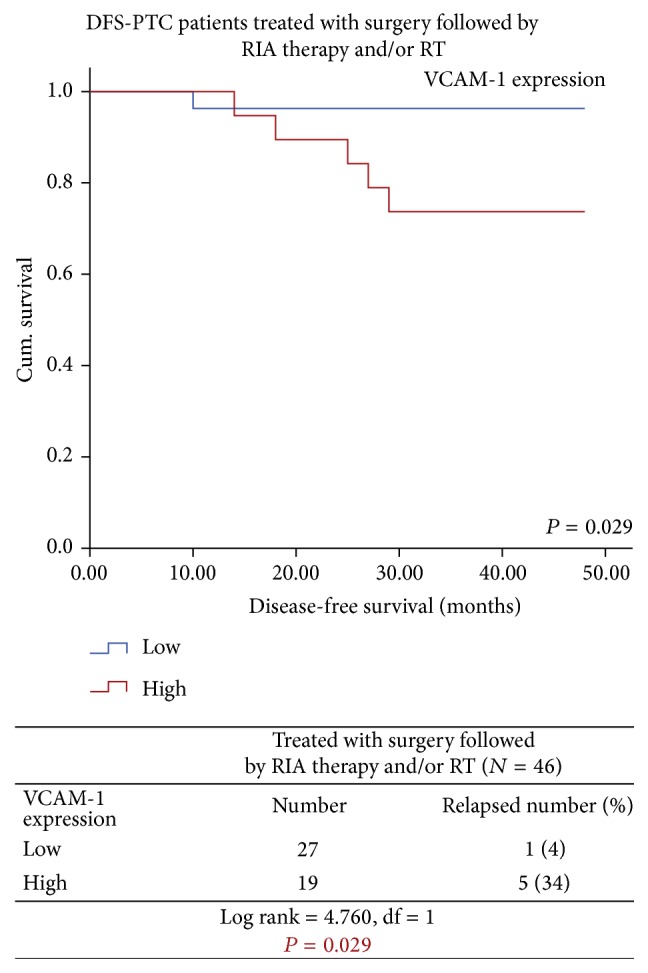
Significantly reduced DFS observed in PTC patients treated with surgery followed by RIA therapy and/or RT having high VCAM-1 expression as compared to those with low VCAM-1 expression.

Moreover, when PTC patients were subgrouped according to the clinicopathological variables, the Kaplan-Meier survival curves demonstrated that high expression of VCAM-1 was remarkably associated with poor DFS in female patients and in the PTC patients postoperatively treated with RIA therapy and/or RT. It was observed that 12% (2/17) of the female PTC patients with high VCAM-1 expression had significantly reduced DFS, whereas none of the female patients with lower VCAM-1 expression had developed recurrence and/or distant metastasis during the follow-up period (Log rank = 3.881, df = 1, *P* = 0.049) (Figure 8) and 34% (5/19) of PTC patients treated with surgery followed by RIA therapy and/or RT having high VCAM-1 expression had reduced DFS in comparison to only 4% (1/27) of these patients with lower VCAM-1 expression (Log rank = 4.760, df = 1, *P* = 0.029) (Figure 9).

On the other hand, the Kaplan-Meier survival analysis and the Log rank test revealed that neither TNF-*α* nor L-Selectin expression emerged as significant prognosticators to predict DFS or OS even when the PTC patients were grouped according to the clinicopathological features.

## 4. Discussion

The current study revealed that serum TNF-*α* was significantly higher in patients with benign thyroid diseases and in PTC patients, as compared to the healthy individuals. ROC curves also validated that TNF-*α* was a potential marker for distinguishing patients with thyroid diseases from the healthy subjects. As compared to the patients with benign thyroid diseases, the circulating levels as well as the tumoural tissue expression of TNF-*α* were significantly higher in PTC patients. The serum TNF-*α* did not show significant correlation with any of the clinicopathological parameters, nor was it associated with DFS in these patients. However, Kaplan-Meier survival analysis revealed that its elevated levels were significantly associated with shorter OS in the PTC patients. But in the multivariate survival analysis, as compared to the clinicopathological prognosticators, it lost its significance as an independent predictor of survival in PTC patients. Results of various other studies also show that, relative to normal healthy controls, a significant increase in circulating levels of TNF-*α* was observed in hepatocellular carcinoma patients [21], cervical neoplasia [22, 23], epithelial ovarian cancer [24, 25], prostate cancer [26], and renal cell carcinoma [27]. In concordance to our observation, high serum TNF-*α* level has been associated with poor prognosis having reduced survival in prostate cancer [26] and epithelial ovarian cancer [25]. Ferrajoli et al. [28] also observed significantly higher plasma TNF-*α* concentration in patients with chronic lymphocytic leukemia than in healthy control population and the elevated circulating TNF-*α* correlated with the extent of disease and was suggested to be a novel prognostic factor for survival in patients with chronic lymphocytic leukemia. Serum TNF-*α* might as well be a risk factor for colorectal cancer [29]. However, Linkov et al. [30] observed that patients with thyroid diseases tended to have lower TNF-*α* levels than the normal reference group, although the difference was not statistically significant and Lumachi et al. [31] have reported that TNF-*α* acts as a growth inhibitor of papillary thyroid cancer cell lines. Agarwal et al. [32] have reported that mean levels of TNF-*α* were also significantly reduced in pretherapeutic serum samples of patients with bladder cancer as compared to the controls, whereas Gendek-Kubiak et al. [33] showed that the average TNF-*α* levels measured in neoplastic patients were not significantly different from that observed in the control group. Differences in results of the reported studies may be related to the difference in the number of patients studied and in their distinct clinical and pathological characteristics.

Further, in the existing study, expression of TNF-*α* was localized in the cytoplasm and was found to be significantly higher in the primary tumours of PTC patients than that observed in patients with benign thyroid diseases. The TNF-*α* expression was significantly positively correlated only with the presence of calcification in the tumours indicating that TNF-*α* is likely to participate in the development of calcification in the tumours. Moreover, tumoural protein expression of TNF-*α* was not able to predict DFS or OS in the PTC patients. In accordance with the present study, cytoplasmic immunoexpression of TNF-*α* has also been reported by De Miguel et al. [34] in epithelial cells of prostate cancer which were increased in comparison to normal prostate tissues. Higher expressions of TNF-*α* have been reported in various tumour tissues [22, 24, 28, 35, 36]. TNF-*α* expression and its action have also been reported in esophageal cancer [37], ovarian cancer [38, 39], breast cancer [40], and follicular thyroid cancer as well [41]. The present study also found a significant inverse correlation between the circulating levels and tumoural protein expression of TNF-*α*. This indicates that the circulating TNF-*α* level was lower in patients having high tumoural expression of this protein and vice versa. Such an inverse correlation can be justified by two probable reasons. Firstly, the absence of shedding-off of the protein overexpressed by tumour cells into the circulation and secondly the higher levels in circulation may be due to stimulation of its production by the immune cells in response to the tumourigenic condition. Thus, the current findings suggest that TNF-*α* could be used as an indicator of thyroid cancer risk from benign conditions and may have a role in development of tumour and prognosis of thyroid cancer patients.

The pretherapeutic circulating levels of both the adhesion molecules L-Selectin and VCAM-1 were significantly higher in patients with the benign disease as well as PTC patients than in the healthy individuals. These results were further confirmed with ROC curve analysis, where both L-Selectin and VCAM-1 could significantly discriminate patients with benign thyroid diseases and PTC from the healthy individuals. However, circulating levels of only L-Selectin, and not of VCAM-1, were significantly elevated in the PTC patients when compared with the benign thyroid disease patients. Similar to these, the results of ROC curves also authenticated the efficacy of L-Selectin to distinguish the PTC patients from those with benign thyroid diseases. Such results confirm essential roles of these adhesion molecules in the development of thyroid tumour.

In accordance with our findings, studies by other authors have also reported significantly higher serum L-Selectin levels in patients with ovarian cancer [42], small-cell and non-small-cell lung cancer [43], chronic myelogenic leukemia [44], and acute myeloid leukemia [45] than those in healthy control groups. Moreover, a study by Chen et al. [46] also demonstrated markedly higher serum levels of L-Selectin in patients with lung cancer or benign diseases than in the healthy controls. They have also reported significant differences in levels of L-Selectin between the lung cancer patients with advanced stage disease or with metastasis and those with early stage disease or with no metastasis. The serum L-Selectin levels were closely related to disease progression in ovarian [42] and liver cancers [47]. In a recent study, circulating L-Selectin levels were also found to be higher in serum samples from patients with high grade metastatic versus high grade nonmetastatic muscle invasive bladder cancer [48]. Moreover, as concerned with the levels of serum VCAM-1, in a study by Pasieka et al. [49], significantly higher levels of VCAM-1 were observed in the serum samples of only anaplastic thyroid carcinoma patients, while in PTC patients the circulating VCAM-1 levels were comparable with those of healthy control group. The increased levels of VCAM-1 in the peripheral blood have also been demonstrated in progression of many forms of cancer: non-small-cell lung cancer [50], breast cancer [17, 51], rectal cancer [52], gastric cancer [53, 54], colorectal cancer [55–59], prostate cancer [60], bladder cancer [61], urological malignancies [62], head and neck cancer [63], pancreatic cancer [64], and ovarian cancer [65]. Recently, Martinez et al. [66] have reported higher levels of VCAM-1 in the bone marrow of advanced breast cancer patients than the healthy volunteers. However, Tas et al. [67] also did not find significant difference in the levels of VCAM-1 between epithelial ovarian cancer patients and control group. Circulating VCAM-1 levels have been found to be associated with more advanced disease in many cancers. Coskun et al. [61] observed that serum VCAM-1 level correlated with tumour stage in bladder cancer patients and was higher in patients with muscle invasive tumours than those with superficial tumours. Alexiou et al. [53] have demonstrated that circulating VCAM-1 was significantly associated with disease stage, gastric wall invasion, lymph node involvement, and distant metastasis in gastric cancer patients. Moreover, in colorectal cancer, it was to be associated with Dukes' D stage and distant metastasis [56]. However, in the present study, serum L-Selectin and VCAM-1 were predominantly positively correlated only with presence of haemorrhagic area and bilaterality of the tumours, respectively, while they were not significantly associated with the rest of the studied clinicopathological parameters in PTC patients. This may be because, in thyroid carcinoma, the risk of disease relapse and occurrence of metastasis is relatively very low as compared to that in other malignancies arising from other organs.

Further, only L-Selectin and not VCAM-1 was significantly overexpressed in the primary tumours of PTC patients when compared to that in patients with benign thyroid diseases. Moreover, L-Selectin immunoreactivity predominantly correlated with larger tumour size and extrathyroidal extension of tumours. Similarly, protein expression of L-Selectin has also been found to be significantly higher in the primary tumours of oral squamous cell carcinoma and salivary gland tumours. Further, in oral squamous cell carcinoma, it was associated with differentiation, TNM stages, and lymph node metastasis [68]. Also in colorectal carcinoma, the L-Selectin overexpression was observed, which was closely associated with the development of the disease and metastasis [69]. Miao et al. [70] also suggested that L-Selectin expression increases in sentinel lymph node metastasis positive breast cancer and that it plays an important role in lymphatic chemotactic metastasis of breast cancer. Moreover, significant attenuation of metastasis was observed in the absence of L-Selectin, indicating that this adhesion molecule actively contributes to leukocyte recruitment and formation of a metastatic niche [71, 72]. Recently, higher expression of L-Selectin in high grade muscle invasive bladder cancer (MIBC) specimens versus low-grade bladder cancer (LGBC) specimens was observed by Choudhary et al. [48]. Further, they observed that L-Selectin localization was seen in foci of metastatic tumour cells in lymph node specimens from patients with high grade MIBC and known nodal involvement.

Cytoplasmic immunoreactivity of VCAM-1 was observed in patients with thyroid diseases. Although not significant, the incidence of VCAM-1 expression was higher in the thyroid cancer patients as compared to the patients with benign thyroid diseases. It has been suggested that proteolytic shedding of VCAM-1 generates its soluble form which can be detected in circulation [73, 74]. In thyroid carcinoma also, there might be shedding of VCAM-1 from the thyroid cancer cells into circulation which may be the factor accounting for the significantly elevated serum levels of VCAM-1. Further, similar to the present study, Huang et al. [75] and Wang et al. [76] have observed cytoplasmic staining of VCAM-1 in ovarian cancer and breast cancer patients, respectively. However, VCAM-1 expression has been found to be overexpressed in primary tumours of many human cancers: ovarian cancer [77], pancreatic cancer [78], gastric cancer [54], oral squamous cell carcinoma [79], and clear cell renal carcinoma as well as papillary renal cell carcinoma [80] as compared to the corresponding noncancerous tissues. Further, in the present study, VCAM-1 expression was significantly overexpressed in the PTC patients with larger tumour size, presence of lymph node metastasis and unifocal tumours, and presence of extrathyroidal extension of tumours as compared to their respective counterparts. Similar to this, VCAM-1 expression was significantly higher in gastric cancer patients with lymph node metastasis than in those without lymph node metastasis [54]. They also demonstrated that VCAM-1 overexpression was associated with clinicopathological stage and depth of infiltration. Moreover, Shin et al. [81] suggested that VCAM-1 expression may contribute to the metastatic adhesion of tumour cells and thus facilitate malignant progression of human gastric tumours. In ovarian cancer, Scalici et al. [77] showed significant increase in VCAM-1 expression with advancing tumour stage and its expression was found to reduce in patients who received neoadjuvant chemotherapy while, in a study by Huang et al. [75], the overexpression of VCAM-1 was associated with advanced age at diagnosis as well as with response to treatment with surgery and chemotherapy. Expression of VCAM-1 was found to be highest in poorly differentiated cutaneous squamous cell carcinoma [82]. Sun et al. [79] documented that, in oral squamous cell carcinoma, VCAM-1 expression was closely correlated to depth of infiltration and lymph node metastasis.

Moreover, in this study, neither the circulating level nor the protein expression of L-Selectin or VCAM-1 was able to predict DFS or OS in total PTC patients. Again such results might be due to better survival and overall very low mortality rates in PTC patients. The results by other authors have suggested that serum L-Selectin could be of prognostic value in ovarian cancer [42] and liver cancer [47] while Li et al. [83] suggested that the detection of protein expression of L-Selectin conduces to judging the prognosis of colorectal cancer patients. But in PTC patients, as the overall mortality is very low, these patients need to be followed up for a longer period of time. Moreover, acute myeloid leukemia patients with higher soluble L-Selectin at diagnosis had high probability of relapse compared to those with normal levels and had shorter event-free survival than patients with lower levels [45].

Although VCAM-1 expression was not able to predict DFS or OS in total PTC patients, Kaplan-Meier survival analysis demonstrated higher incidence of disease relapse in patients with high VCAM-1 expression as compared to those with low VCAM-1 expression. Moreover, higher VCAM-1 expression was significantly associated with the reduced DFS in female PTC patients and in those who were postoperatively treated with RIA and/or RT. Thus, it can be suggested that the higher VCAM-1 expression in the primary tumours may be helpful in predicting DFS in female PTC patients and also the extent of treatment that might be required in the PTC patients with VCAM-1 overexpression. However, although not independent of tumour stage, serum VCAM-1 levels were significant prognostic factors for patient survival in gastric cancer [53] and colorectal cancer [55]. Moreover, serum VCAM-1 was the only prognostic factor for patients with stage 3 and stage 4 rectal cancer patients [52] while, in prostate cancer, serum VCAM-1 achieved the status of independent predictor after adjusting for the standard postoperative clinicopathological features [60]. Ho et al. [84] observed better DFS in hepatocellular carcinoma patients with low VCAM-1 levels. The serum VCAM-1 levels were also found to have prognostic significance in breast cancer patients [51, 85]. Further, expression of VCAM-1 in high grade serous ovarian cancer was associated with poor prognosis [75].

There was a significant positive correlation between circulating L-Selectin and TNF-*α* in the studied PTC patients, while VCAM-1 expression was predominantly and positively associated with TNF-*α* immunoreactivity. This is in relation to Borsig [86], who suggested that selectins may be expressed and/or activated in presence of certain mediators like TNF-*α*, interleukins, or other toxins. Radhakrishnan et al. [87] showed that TNF-*α* enhances the motility and invasiveness of human prostate cancer cells by stimulating the expression of selective glycosyl- and sulfotransferase genes involved in the synthesis of selectin ligands. Further, they suggested that interactions of selectins and their ligands play a crucial role in enhancing the potential of the cancer cells to target to the lymphoid organs and inflamed endothelium at distant sites. Moreover, there are also studies indicating that TNF-*α* produced by stimulated Kupffer cells appeared to promote expression of adhesion molecules early during the metastatic process [88–90]. In breast cancer cell lines, Ali et al. [91] observed that VCAM-1 expression was enhanced by treatment with TNF-*α*.

Thus, collectively these results support the idea that host responses to cancer cells may cause expression of adhesion molecules via the production of inflammatory cytokine like TNF-*α*, which can facilitate tumour promotion and progression and further substantiate the link between inflammation and cancer progression.

## 5. Conclusion

The results of the present study signify a probable role of TNF-*α* and the adhesion molecules: L-Selectin and VCAM-1 in thyroid carcinogenesis. Determining their circulating levels may serve as a promising noninvasive method to uncover diagnostically informative differences between benign and malignant thyroid conditions. This may further aid in the results of indeterminate fine needle aspiration results and also in the preoperative prediction of malignancy in patients with nodular thyroid diseases. On the whole, the interaction between TNF-*α* and the adhesion molecules may form a comprehensive network which may induce sustained activation of various signalling pathways in follicular cells. Thus, understanding this complexity may offer potential therapeutic targets for management of thyroid cancer which may help the clinicians to identify and perform more personalized approach to their patients with more aggressive tumours, sparing the vast majority of patients with indolent disease from unnecessary procedures. Developing preventive and therapeutic strategies targeted towards TNF-*α* may help induce antitumour immunity, further reducing the rates of recurrence and mortality in thyroid cancer.

## Figures and Tables

**Table 1:** Clinicopathological characteristics of PTC patients.

Characteristics	*N* (%)	Characteristics	*N* (%)
Age		Bilaterality	
<45 years	41 (49)	Unilateral	61 (74)
≥45 years	42 (51)	Bilateral	22 (26)
Gender		Haemorrhagic area	
Female	56 (68)	Absent	72 (87)
Male	27 (32)	Present	11 (13)
Tumour size		Necrosis	
T1 (*N* = 16) + T2 (*N* = 22)	38 (46)	Absent	67 (81)
T3 (*N* = 30) + T4 (*N* = 15)	45 (54)	Present	16 (19)
Nodal status		Calcification	
Absent	30 (36)	Absent	32 (39)
Present	53 (64)	Present	51 (61)
Metastasis		Extrathyroidal extension	
Absent	73 (88)	Absent	52 (63)
Present	10 (12)	Present	31 (37)
Stage		Fibrosis	
Early (Stage I (*N* = 37) + Stage II (*N* = 12))	49 (59)	Absent	61 (74)
Advanced (Stage III (*N* = 11) + Stage IV (*N* = 23))	34 (41)	Present	22 (26)
Lymphatic permeation		Inflammation	
Absent	67 (81)	Absent	46 (55)
Present	16 (19)	Present	37 (45)
Vascular permeation		Differentiation	
Absent	74 (89)	Well	76 (92)
Present	09 (11)	Moderate/poor	07 (08)
Capsular invasion		Multifocality	
Absent	55 (66)	Absent	64 (77)
Present	28 (34)	Present	19 (23)
Encapsulation		Residual disease	
Well encapsulated	76 (92)	Absent	24 (29)
Partially/not encapsulated	07 (08)	Present	59 (71)

Treatment			
Surgery	29 (35)		
Surgery + RIA and/or RT	54 (65)	Surgery + RIA	50 (60)
		Surgery + RIA + RT	04 (05)

Disease status			
Recurrence/distant metastasis (*N* = 69)		Alive/dead (*N* = 76)	
Absent	62 (90)	Alive	68 (89)
Present	07 (10)	Dead	08 (11)
Recurrence	3 (4)		
Distant metastasis	4 (6)		
Bone	1 (1.5)		
Lung	2 (3.0)		
Bone + lung	1 (1.5)		

**Table 2:** Significance of circulating levels of TNF-*α*, L-Selectin, and VCAM-1 in patients with benign thyroid diseases and PTC.

Subjects	TNF-*α*		L-Selectin		VCAM-1	
	M ± SE (pg/mL)	*P*	M ± SE (pg/mL)	*P*	M ± SE (pg/mL)	*P*
Healthy individuals (*N* = 67)	2.16 ± 0.37		11.87 ± 4.38		161.90 ± 30.02	
Benign thyroid diseases (*N* = 67)	14.45 ± 1.95	<0.001^*∗*^	2064.13 ± 104.09	<0.001^*∗*^	771.48 ± 42.83	<0.001^*∗*^
Papillary thyroid carcinoma (*N* = 83)	49.37 ± 11.73	<0.001^†^	3028.03 ± 202.26	<0.001^†^	777.91 ± 39.36	<0.001^†^
		0.009^‡^		<0.001^‡^		0.912^‡^

^**∗**^Significance between benign thyroid diseases and healthy individuals.

^†^Significance between PTC and healthy individuals.

^‡^Significance between PTC and benign thyroid diseases.

**Table 3:** Correlation of circulating levels of TNF-*α*, L-Selectin, and VCAM-1 with clinicopathological parameters of PTC patients.

Parameter	*N*	TNF-*α*	*P*	L-Selectin	*P*	VCAM-1	*P*
		Mean ± SE		Mean ± SE		Mean ± SE	
Bilaterality							
Unilateral	61	56.02 ± 15.54	0.349	2980.28 ± 206.67	0.697	728.73 ± 42.97	0.037
Bilateral	22	30.94 ± 9.57		3160.43 ± 512.96		914.27 ± 83.66	
Haemorrhagic area							
Absent	72	52.89 ± 13.46	0.446	2827.29 ± 174.54	0.010	802.79 ± 43.32	0.106
Present	11	26.30 ± 5.74		4341.93 ± 957.44		615.04 ± 97.87	
Calcification							
Absent	32	23.50 ± 6.76	0.081	2825.51 ± 263.16	0.431	843.05 ± 86.75	0.192
Present	51	65.60 ± 18.33		3155.10 ± 285.32		737.04 ± 33.50	
Extrathyroidal extension							
Absent	52	32.67 ± 11.70	0.065	2968.66 ± 238.59	0.706	746.04 ± 39.64	0.297
Present	31	77.37 ± 23.99		3127.61 ± 369.48		831.38 ± 81.90	

**Table 4:** Comparison of expressions of TNF-*α*, L-Selectin, and VCAM-1 between the patients with benign thyroid diseases and PTC.

Patients		TNF-*α*		L-Selectin		VCAM-1	
	*N* (%)	Low	High	Low	High	Low	High
		*N* (%)	*N* (%)	*N* (%)	*N* (%)	*N* (%)	*N* (%)
Benign thyroid diseases	45	36 (80)	9 (20)	37 (82)	8 (18)	32 (71)	13 (29)
PTC	83	46 (55)	37 (45)	52 (63)	31 (37)	49 (59)	34 (41)
		*χ* ^2^ = 7.657, *r* = +0.245, *P* = 0.005		*χ* ^2^ = 5.276, *r* = +0.203, *P* = 0.022		*χ* ^2^ = 1.831, *r* = +0.120, *P* = 0.179	

**Table 5:** Correlation of tumoural protein expression of TNF-*α*, L-Selectin, and VCAM-1 with clinicopathological parameters of PTC patients.

		TNF-*α*		L-Selectin		VCAM-1	
Parameter	*N*	Low	High	Low	High	Low	High
		*N* (%)	*N* (%)	*N* (%)	*N* (%)	*N* (%)	*N* (%)
Tumour size							
Small (T1 + T2)	38			30 (79)	8 (21)	28 (74)	10 (26)
Large (T3 + T4)	45	—		22 (49)	23 (51)	21 (47)	24 (53)
				*χ* ^2^ = 7.955, *r* = +0.310, *P* = 0.004		*χ* ^2^ = 6.219, *r* = +0.274, *P* = 0.012	

Nodal status							
N0	30					22 (73)	8 (27)
N1	53	—		—		27 (51)	26 (49)
						*χ* ^2^ = 3.971, *r* = +0.219, *P* = 0.047	

Multifocality							
Absent	64					34 (53)	30 (47)
Present	19	—		—		15 (79)	4 (21)
						*χ* ^2^ = 4.040, *r* = −0.221, *P* = 0.045	

Calcification							
Absent	32	23 (72)	9 (28)				
Present	51	23 (45)	28 (55)	—		—	
		*χ* ^2^ = 5.706, *r* = +0.262, *P* = 0.017					

Extrathyroidal extension							
Absent	52			40 (77)	12 (23)	38 (73)	14 (27)
Present	31	—		12 (39)	19 (61)	11 (35)	20 (65)
				*χ* ^2^ = 12.120, *r* = +0.382, *P* < 0.001		*χ* ^2^ = 11.350, *r* = +0.370, *P* = 0.001	

**Table 6:** Correlation of TNF-*α* with L-Selectin and VCAM-1 in PTC patients.

	L-Selectin	VCAM-1	
TNF-*α*	Circulating levels		*r* = −0.254, *P* = 0.021^*∗*^
	*r* = +0.303, *P* = 0.005	*r* = +0.011, *P* = 0.923	
	Tumoural protein expression		
	*r* = +0.036, *P* = 0.745	*r* = +0.347, *P* = 0.001	

^*∗*^Correlation between circulating and tumoural protein expression of TNF-*α*.

**Table 7:** Univariate survival analysis for DFS in PTC patients (*N* = 69).

Variables	*N*	Patients relapsed	Log rank test statistics
		*N* (%)	
Gender			
Female	49	2 (4)	Log rank = 7.107, df = 1, *P* = 0.008
Male	20	5 (25)	
Encapsulation			
Well encapsulated	63	5 (8)	Log rank = 4.227, df = 1, *P* = 0.040
Partially/not encapsulated	6	2 (33)	

Circulating levels			
Circulating TNF-*α*			
Low	37	5 (13)	Log rank = 0.999, df = 1, *P* = 0.318
High	32	2 (6)	
Circulating L-Selectin			
Low	33	4 (12)	Log rank = 0.254, df = 1, *P* = 0.614
High	36	3 (8)	
Circulating VCAM-1			
Low	35	4 (11)	Log rank = 0.082, df = 1, *P* = 0.775
High	34	3 (9)	

Tumoural protein expression			
TNF-*α* expression			
Low	41	6 (15)	Log rank = 2.147, df = 1, *P* = 0.143
High	28	1 (4)	
L-Selectin expression			
Low	41	3 (8)	Log rank = 0.456, df = 1, *P* = 0.500
High	28	4 (12)	
VCAM-1 expression			
Low	42	2 (5)	Log rank = 3.415, df = 1, *P* = 0.065
High	27	5 (18)	

**Table 8:** Univariate survival analysis for OS in PTC patients (*N* = 69).

Variables	*N*	Patients died	Log rank test statistics
		*N* (%)	
Age			
<45 years	37	1 (3)	Log rank = 4.472, df = 1, *P* = 0.034
≥45 years	39	7 (18)	
Gender			
Female	51	2 (4)	Log rank = 6.870, df = 1, *P* = 0.009
Male	25	6 (24)	
Metastasis			
Absent	67	5 (7)	Log rank = 7.581, df = 1, *P* = 0.006
Present	9	3 (33)	
Stage			
Early (I + II)	43	1 (2)	Log rank = 6.859, df = 1, *P* = 0.009
Advanced (III + IV)	33	7 (21)	
Multifocality			
Absent	59	4 (7)	Log rank = 4.130, df = 1, *P* = 0.042
Present	17	4 (23)	

Circulating levels			
Circulating TNF-*α*			
Low	38	1 (3)	Log rank = 5.129, df = 1, *P* = 0.024
High	38	7 (18)	
Circulating L-Selectin			
Low	38	5 (13)	Log rank = 0.502, df = 1, *P* = 0.479
High	38	3 (8)	
Circulating VCAM-1			
Low	38	3 (8)	Log rank = 0.515, df = 1, *P* = 0.473
High	38	5 (13)	

Tumoural protein expression			
TNF-*α* expression			
Low	44	5 (11)	Log rank = 0.059, df = 1, *P* = 0.808
High	32	3 (91)	
L-Selectin expression			
Low	46	7 (15)	Log rank = 2.697, df = 1, *P* = 0.101
High	30	1 (7)	
VCAM-1 expression			
Low	46	5 (11)	Log rank = 0.027, df = 1, *P* = 0.870
High	30	3 (10)	
